# Effective separation of protein from *Polygonatum cyrtonema* crude polysaccharide utilizing ionic liquid tetrabutylammonium bromide

**DOI:** 10.3389/fchem.2023.1287571

**Published:** 2024-01-08

**Authors:** Yuling Xu, Jing Xu, Zheng Fan, Siyuan Zhang, Yuanjie Wu, Rongchun Han, Nianjun Yu, Xiaohui Tong

**Affiliations:** ^1^ School of Pharmacy, Anhui University of Chinese Medicine, Hefei, China; ^2^ School of Life Sciences, Anhui University of Chinese Medicine, Hefei, China; ^3^ Medical Department, Taihe Hospital of Chinese Medicine, Taihe, China; ^4^ School of Traditional Chinese Medicine, Anhui University of Chinese Medicine, Hefei, China; ^5^ Joint Research Center for Chinese Herbal Medicine of Anhui of IHM, Anhui University of Chinese Medicine, Hefei, China

**Keywords:** *Polygonatum cyrtonema*, tetrabutylammonium bromide, aqueous two-phase system, polysaccharide, ionic liquid

## Abstract

Extraction of plant polysaccharides often results in a large amount of proteins, which is hard to eliminate from the crude extract, and conventional approaches for deproteinization are time-consuming and often involve hazardous organic solvents. In this study, ionic liquid tetrabutylammonium bromide (TBABr) was used to create an ionic liquid aqueous two-phase system (ILATPS) for the separation of the polysaccharide (PcP) and protein extracted from the rhizome of *Polygonatum cyrtonema*. Bovine serum albumin (BSA) was first applied to assess the feasibility of the ILATPS, and MgSO_4_ was determined to be the most suitable inorganic salt. By adopting the Taguchi experiment with an L9 (3^4) orthogonal array, it was found that the best condition for the efficient separation of crude PcP was at 25°C, with 1.5 g of TBABr, 15 mg of PcP, and 2.0 g of MgSO_4_, with the extraction efficiency for the protein and polysaccharide as 98.6% and 93.5%, respectively. The purified PcP was homogeneous, and its weight average molecular weight (Mw) was 7,554 Da. Monosaccharide composition analysis indicated the PcP comprised mannose, galactose, glucose, galacturonic acid, arabinose, and rhamnose at a molar ratio of 33:13:8:3.5:2:1. This approach offers a practical tactic to purify polysaccharides of plant origin.

## 1 Introduction

Existing in the form of branched or linear glycosidic chains, natural polysaccharide (PcP) belongs to a type of macromolecular polymer with over 10 covalently bonded monosaccharides and a molecular weight ranging from thousands to several million Daltons (Yu et al., 2018; Ji et al., 2020). According to distinct sources, it can be categorized into microbial, animal, and plant polysaccharides, which provides energy and nutrition, such as starch and glycogen, or exerts specific functions like cellulose, pectin, and chitin. From the perspective of plant physiology, polysaccharide is essential in helping plants overcome difficult conditions including drought and cold. Moreover, it is also crucial for general life activities, for example, cell communications, molecular recognition, and cell adhesion (Dwek, 1996). In the past decades, more and more attention was drawn to versatile pharmacological properties of polysaccharides derived from natural sources. Polysaccharides purified from fungi, seaweeds, higher plants, and insects demonstrated antioxidant, antiviral, immunomodulatory, antitumor, and hepatoprotective effects (Cho et al., 2015; Choromanska et al., 2015; Qu et al., 2020). The polysaccharide extracted from *Gracilaria rubra* and *Ginkgo biloba* exhibited immunostimulating and antioxidant activities (Di et al., 2017; Ren et al., 2019), while the polysaccharide extracted from *Astragalus* was reported to possess anti-diabetic activity and improve cognitive dysfunction by altering the gut microbiota in a rodent model (L et al., 2019). In particular, the polysaccharide extracted from *Polygonatum cyrtonema* Hua demonstrated anti-fatigue effects by regulating the osteocalcin signaling pathway and modulating osteocalcin-mediated crosstalk-linking muscles and bones (Shen et al., 2021; Li et al., 2023).

On one hand, the pharmacological properties of the polysaccharide rely on its physicochemical and structural characteristics comprising the solubility, molecular weight, molecular structure, monosaccharide species, and degree of branching (Yang et al., 2019); on the other hand, to extract a polysaccharide of high quality without sabotaging its structure, technical barriers should be resolved. Extracting the polysaccharide is relatively easy because it normally has satisfactory water solubility. However, removing interfering substances from the crude polysaccharide is challenging as the extracted small molecules and proteins with similar physical properties are difficult to remove. A case in point is that despite numerous studies documenting the tangible efficacy of the polysaccharide in treating various diseases, a number of medicines in domestic and international markets are very much limited, at least in part due to considerable complexity involving extraction and purification (Yang et al., 2022).

By combining ionic liquid and water to form an aqueous two-phase system (ATPS), this liquid–liquid fractionation approach offers a new solution (Antunes et al., 2023). Compared with conventional methods, ATPS excels because it is capable of continuous operation, easy to scale up, and environmentally friendly (Ruiz-Ruiz et al., 2012; Li et al., 2017). A typical ionic liquid consists of a large and unsymmetrical organic cation and an inorganic or organic anion. In addition, it is a molten salt with a melting temperature often below 100°C. One prominent feature of the ionic liquid is its extraordinary ability to dissolve a wide range of chemicals and is an ideal stabilizing solvent for proteins (Iqbal et al., 2016). Removing proteins is usually the most important operation, and before the advent of the ionic liquid, deproteinization involves organic solvents which are often hazardous. For example, chloroform-n-butanol used in the Sevag reagent denatures and precipitates proteins between the organic and water layers, and the pellet will be removed (Chen et al., 2021). The Sevag approach uses large amounts of organic solvents due to repetitive operations, while the extraction efficiency is sometimes low.

In this study, ionic liquid tetrabutylammonium bromide (TBABr) was added to a water solution containing MgSO_4_ to prepare an ionic liquid aqueous two-phase system (ILATPS), which was used to separate the PcP and protein extracted from the rhizome of *P*. *cyrtonema*, a frequently prescribed traditional Chinese medicine with the polysaccharide as its key active component. The composition and preliminary structure of the purified PcP were determined as well. It was found that under optimal conditions, the ILATPS featuring TBABr and MgSO_4_ is suitable for the effective separation of the PcP and protein in *P. cyrtonema*.

## 2 Materials and methods

### 2.1 Materials and reagents

Four-year-old rhizomes of *P. cyrtonema* were collected from Qingyang County, Anhui Province, China, in July 2022. Fresh samples were sliced and freeze-dried using an LGJ-10 lyophilizer (Songyuan, China) and then subjected to grinding. Fine powders that could pass through a 50-mesh sieve were used for the extraction of the crude PcP. The chemicals and reagents of high purity utilized in this study are given in Table 1. All other reagents including MgSO_4_, K_3_PO_4_, KOH, Na_2_CO_3_, K_2_HPO_4_, and CaCl_2_ were analytically pure.

**TABLE 1:** Chemicals used for the experiments and their specifications.

Name of the chemical	Specification	Manufacturer
Tetrabutylammonium bromide (TBABr)	99% purity	Merck (St. Louis, United States)
Dextran	1, 5, 12, 25, 50, 80, 150, and 270 kDa, all 97% purity	Merck (St. Louis, United States)
Monosaccharide	Mannose, D-glucosamine HCl, rhamnose, glucuronic acid, galacturonic acid, D-galactosamine HCl, glucose, galactose, xylose, arabinose, and fucose, all 98% purity	Merck (St. Louis, United States)
Bovine serum albumin (BSA)	99% purity	Macklin (Shanghai, China)
Coomassie brilliant blue	90% purity	Macklin (Shanghai, China)

### 2.2 Extraction of the crude PcP

Approaches including water, enzymatic, ultrasonic, microwave, and supercritical fluid extraction were applied to obtain plant polysaccharides, and evidence suggested hot water extraction using ultrasonic waves may yield good results (Fan et al., 2022). Such a procedure includes the following steps: cleaning, smashing, extraction, precipitation, and drying (Liu et al., 2019). Hot water and the powdered raw material in a fixed ratio are used for extracting the crude polysaccharide, which will be presented in the supernatant. Then, a certain amount of ethanol is mixed with the supernatant to produce the precipitate that contains the polysaccharide, unwanted protein, and other small molecules. For the determination of its structure and the related physiological or pharmacological function, the polysaccharide must be purified before the steps including dialysis, gel filtration, and infrared chromatography can be applied.

In this study, to remove lipids and pigments, rhizome powder (8 g) of *P. cyrtonema* was subjected to refluxing extraction for 3 h by using petroleum ether. The residue was freeze-dried and added to 240 g of double-distilled water (ddH_2_O) for ultrasonic extraction at 60°C for 1 h. Suction filtration was subsequently applied to collect the supernatant, which was then subjected to ethanol precipitation (Liu and Huang, 2019). For every 100 mL of the supernatant, 400 mL of absolute ethanol was used. The mixture was maintained at 4°C for 12 h before centrifugation at 10,000 × g for 15 min, and the precipitate was dialyzed in ddH_2_O for 48 h (Samrot et al., 2020). The resultant pellet was finally freeze-dried as the crude PcP. The color of the crude PcP was pale. By using Coomassie brilliant blue G-250 and the anthrone–sulfuric acid colorimetric method, the contents of the protein and carbohydrate were found to be 7.1% and 54.3%, respectively.

### 2.3 Separation of the PcP and protein using the ILATPS

Phase separation, a prerequisite for the ILATPS, is affected by the types of inorganic salts, and in this study, MgSO_4_, K_3_PO_4_, K_2_HPO_4_, NaHCO_3_, KOH, Na_2_CO_3_, CaCl_2_, NaCl, and KCl were utilized. After 1.75 g of designated inorganic salt was dissolved in 5 mL of ddH_2_O, 1.0 g of TBABr was added to the solution, which was mixed thoroughly for 5 min using a vortex mixer. The solution was allowed to stand for 30 min, and the salts that caused phase separation were used for the subsequent steps.

To assess the ability of TBABr in extracting protein from an aqueous solution, different combinations of the ionic liquid and competent inorganic salts were applied. For every combination, 50 μL of the BSA solution with a concentration of 200 μg/μL was mixed with 4,950 μL of ddH_2_O containing 1.75 g of inorganic salt; then, 1.0 g of TBABr was added in a 15-mL centrifuge tube. The respective blank controls including all ingredients but BSA were set for calibration. Before centrifugation at 10,000 × g for 15 min to form the lower aqueous phase and the upper phase comprising mostly TBABr, the solutions were mixed well and then incubated at room temperature for 30 min. The aqueous phase was separated using a pipette, with the respective volume being documented. The amount of BSA left in the lower phase was measured using the Bradford method (Bradford, 1976) for the calculation of the extraction efficiency of TBABr.

With an optimal combination of the ionic liquid and inorganic salt in hand, BSA was replaced by crude PcP for downstream analysis. To achieve high extraction efficiency, four variables at three different levels were designed. A full factorial trial would require (3^4) = 81 experiments. We designed a Taguchi experiment with an L9 (3^4) orthogonal array (9 tests, 4 variables, and 3 levels). The orthogonal array for this study is given in Table 2. Different parameters including the concentration of the inorganic salt, concentration of the ionic liquid, quantity of crude PcP in the system, and temperature were taken into consideration.

**TABLE 2:** Taguchi L9 orthogonal array with test results.

No.	Inorganic salt (%)	IL (%)	PcP (mg)	Temperature (°C)	Protein extraction (%)	Sugar remaining (%)
1	30	30	5	25	97.5	92.4
2	30	35	10	35	98.3	58.4
3	30	40	15	45	99.0	51.7
4	35	30	10	45	98.9	53.8
5	35	35	15	25	98.6	88.1
6	35	40	5	35	97.9	59.3
7	40	30	15	35	98.4	72.7
8	40	35	5	45	98.2	50.2
9	40	40	10	25	98.8	87.2

The signal-to-noise (S/N) ratio analysis, as well as the analysis of variance, was carried out to obtain the best combination of parameters, as well as the percentage contribution of each parameter (Pruthvi et al., 2016). By this means, we determined the optimal process parameters which were applied to produce the purified PcP. The aqueous phase containing the polysaccharide was dialyzed in deionized water for 24 h and then precipitated using absolute ethanol. The pellet was freeze-dried to produce purified PcP for the following physical and chemical analyses.

### 2.4 Determination of the polysaccharide and protein concentration in the ILATPS

The possibility that the separated carbohydrate and protein might not be intact due to the pyrolysis process should be considered. According to Chen et al. (2018), thermal degradation of carbohydrate and protein in three species of microalgae takes place in temperature ranges of 164–497 and 209°C–309°C. Although the detailed pyrolysis data on *P*. *cyrtonema* are currently not available, both refluxing and ultrasonic extraction under 60°C will not have a significant impact on their respective structures. The quantification of both the polysaccharide and protein in crude PcP was undertaken using anthrone–sulfuric acid and the Bradford method (Bradford, 1976). For polysaccharide assessment, after the preparation of a series of reference solutions using anhydrous glucose, a 0.2% solution of anthrone in sulfuric acid was added to the respective solutions, whose absorbance at 582 nm was then measured to plot the standard curve. *Solomon seal* polysaccharide, calculated as anhydrous glucose, was subsequently determined (Pharmacopoeia, 2020). The formulas used to calculate the extraction efficiency of carbohydrate and protein in crude PcP, and the adjusted preparation procedure for the Coomassie brilliant blue G-250 solution were followed according to Yang et al. (2022).

### 2.5 Analyses of purified PcP homogeneity and molecular weight

High-performance gel-permeation chromatography (HPGPC) was utilized to determine the homogeneity and molecular weight of purified PcP on a Waters 1515 gel permeation chromatography platform coupled with an OHpak SB-804 HQ column (Shodex, Japan) and a Waters 2410 refractive index detector (Waters, United States). Dextran standards with the following molecular weights were used to establish a calibration curve: 1, 5, 12, 25, 50, 80, 150, and 270 kDa. The purified PcP was dissolved in the NaCl solution (0.05 M) to obtain a final concentration of 5 mg/mL and then filtered for HPGPC analysis. Chromatographic conditions were set as follows: NaCl solution (0.05 M) as the mobile phase with the column temperature set to 40°C; a 65-min isocratic elution with a flow rate of 0.65 mL/min; and injection volume 30 μL.

### 2.6 PcP monosaccharide composition analysis

Next, 5 mg of purified PcP was added to 1 mL of the 2.0 M trifluoroacetic acid (TFA) solution and kept in a sealed container at 121°C for 2 h for hydrolysis. The hydrolyzed sample was then dried on a nitrogen blowing instrument. After the residue was rinsed with methanol three times and dried under the protection of nitrogen, it was dissolved in ddH_2_O for 1-phenyl-3-methyl-5-pyrazolone (PMP) derivatization. The sample and monosaccharide standards were analyzed on an U3000 HPLC system (Thermo Fisher, United States) equipped with a ZORBAX Eclipse XDB-C18 column, and the column temperature was set to 30°C. Chromatographic conditions were set as follows: A solvent of acetonitrile and B solvent of sodium phosphate buffer (pH = 6.8) at the ratio of 17: 83 were used to produce a 60-min isocratic elution at a flow rate of 0.8 mL/min, with an injection volume of 10 μL and detection wavelength 250 nm. The respective chromatographic peaks of monosaccharide standards were manually integrated, and sugar concentrations in the sample were quantified using an external calibration curve.

### 2.7 Determination of PcP infrared spectra

A measure of 2 mg of purified PcP was added to 200 mg of KBr, and the mixture was ground extensively. A disk was then prepared by pressing the mixture for analysis. Fourier-transform infrared (FT-IR) spectra of PcP with a wavelength ranging from 4,000 to 400 cm^−1^ were determined using a Nicolet 6700 spectrometer (Thermo Fisher, United States)

### 2.8 Data analysis

The Taguchi method was designed using Minitab 21.1 software. For a larger-the-better quality characteristic, the S/N ratio, which minimizes the effect of noise on the response, was computed using the following equation: S/N = −10 × log_10_ (∑(1/Y^2^)/n), where n denotes the number of observations in the factor-level combination and Y denotes the obtained responses for the given factor-level combination. GraphPad Prism 7.00 software was used to process the observed parameters.

## 3 Results and discussion

### 3.1 Optimal conditions for the ILATPS

#### 3.1.1 Determination of suitable inorganic salts using the BSA solution

Addition of an ionic liquid to water does not automatically form two phases, which are guaranteed by the right type of inorganic salt. For the current study, nine inorganic salts were tested. Concerning their ability for phase separation, MgSO_4_, K_3_PO_4_, K_2_HPO_4_, KOH, and Na_2_CO_3_ were applicable, while the addition of CaCl_2_, NaCl, and KCl did not cause phase separation, and NaHCO_3_ resulted in precipitation. By reducing the quantity from 1.75 g to 1.0 g for all salts, we observed similar outcomes, except that K_2_HPO_4_ at a lower concentration failed in separating the phases. Mechanistically, certain anions such as OH^−^, SO_4_^2−^, CO_3_^2−^, and PO_4_^3−^ facilitate phase separation in the ILATPS because of their strong acting forces with H_2_O. The salt ion charge plays an essential role that is responsible for the formation of hydration complexes (Yang et al., 2018). Regarding the interactions between TBABr and inorganic salts, the Gibbs free energy of hydration (ΔG_hyd_) also contributes to the successful formation of two layers (Yan et al., 2014). Concerning ΔG_hyd_, PO_4_^3−^ > CO_3_^2−^ > OH^−^, and this order is in line with the Hofmeister series for the strength of the kosmotropic salts (Pei et al., 2009).

**FIGURE 1:**
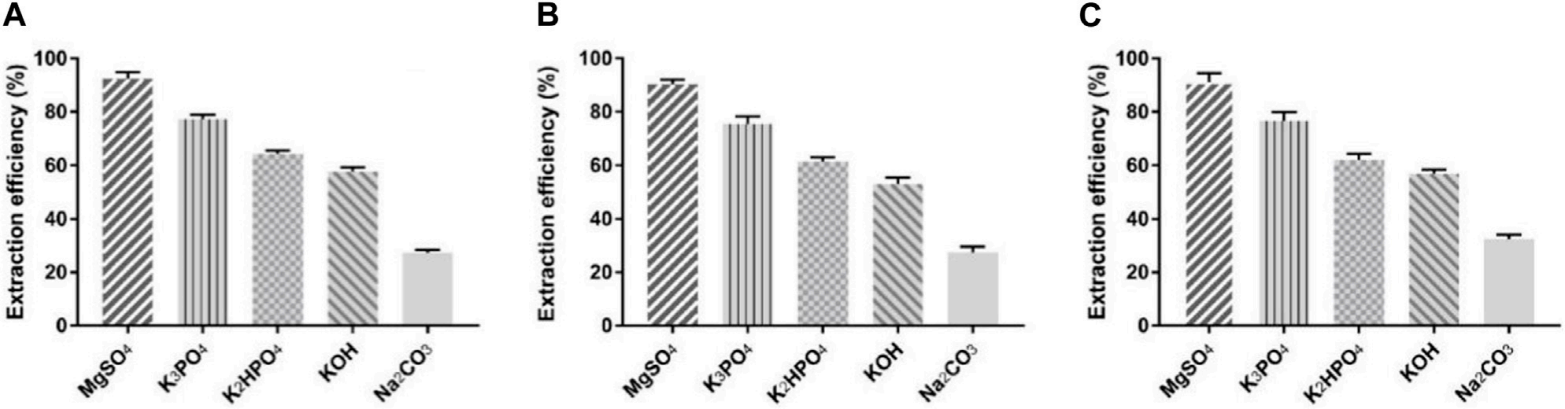
BSA extraction efficiency using TBABr and five inorganic salts. **(A)** 1.75 g of TBABr used; **(B)** 1.5 g of TBABr used; **(C)** 2.0 g of TBABr used. Data presented are the mean with SD and *n* = 3 per group.

As a good starting material to test the protein partitioning capability in the ILATPS, BSA was trialed in several ionic liquid systems including [C_4_mim]Cl, [C_8_mim]Br, and Ammoeng 110, and parameters including the concentration of ILs, pH value, and temperature were also analyzed (Pei et al., 2009). We used 5.0 mL of ddH_2_O containing 10 mg of BSA, 1.75 g of inorganic salts, and 1.0 g of TBABr to assess its extraction ability at room temperature and found that MgSO_4_ performed the best in terms of protein extraction, followed by K_3_PO_4_, K_2_HPO_4_, KOH, and Na_2_CO_3_ (Figure 1A). All the five inorganic salts helped TBABr extract BSA to a certain extent, with MgSO_4_ achieving an extraction rate of 92.4% (Figure 1A). For comparison, 1.5 g and 2.0 g of respective inorganic salts were also trialed using the same system. It was found that MgSO_4_ was the most efficient salt facilitating the extraction of BSA. With 1.5 g of MgSO_4_ used, the average extraction rate was 90.3% (Figure 1B), and 2.0 g of MgSO_4_ resulted in an average extraction rate of 91.1% (Figure 1C). Hence, MgSO_4_ was selected as the salting-out chemical for downstream studies. It appeared that different types of salts influenced the extraction efficiency more than the quantity of salts used.

#### 3.1.2 Determination of optimal parameters using the Taguchi method

We considered four variables that might affect the extraction efficiency of the protein and polysaccharide in crude PcP. The concentration of MgSO_4_ in the ILATPS was set to 30%, 35%, and 40% (*w*/*v*); the concentration of TBABr was set to 30%, 35%, and 40% (*w*/*v*); the amount of crude PcP used was 5 mg, 10 mg, and 15 mg; and temperatures were controlled at 25°C, 35°C, and 45°C (Table 2). It is time-consuming to test all the 81 combinations, and it is not necessary because the experimental design Dr. Genichi Taguchi developed allows the use of orthogonal arrays to evaluate variables and the associated levels systematically for the purpose of completing the experiment with minimum trials. In our case, nine experiments were set up to find the best conditions.

**FIGURE 2:**
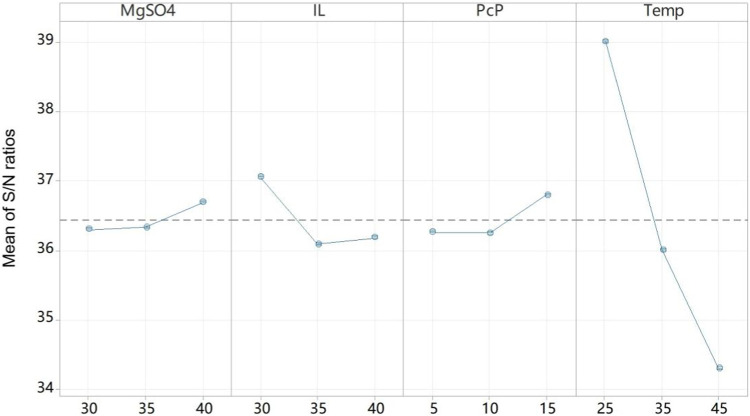
Main effect plot for S/N ratios. Signal to noise: larger is better.

The protein extraction rate for the nine representative combinations given in Table 2 ranged from 97.5% to 99.0%, all suggesting fairly good results in terms of eliminating the protein from crude PcP. The S/N ratio was computed for protein extraction efficiency as the response using Minitab 21.1, and the data indicated increasing temperature and quantities of MgSO_4_ and PcP, as well as TBABr, were generally applicable to achieve an even better extraction rate. However, judging from Table 2, the amount of polysaccharide that was left in the aqueous phase, following ILATPS separation, varied drastically from 50.2% to 92.4%. Therefore, we focused on factors that could influence PcP partition in the ILATPS. Minitab provides four S/N ratios to deal with the respective goals of the experiments: larger is better, smaller is better, nominal is best (Ⅰ), and nominal is best (Ⅱ) (Osman, 1997). Since keeping as many polysaccharides as possible in the aqueous phase is the desired status, the option of larger is better was chosen for this experiment. As a result, the analysis of the S/N ratios led to the conclusion that the factors in consideration exhibited an increase in keeping the polysaccharide in the aqueous phase with increased levels of MgSO_4_ and PcP (Figure 2). On the other hand, higher temperature and larger quantity of the ionic liquid sabotaged the partitioning capability of the ILATPS, leading to the relocation of the polysaccharide in TBABr (Figure 2). According to the response values for the S/N ratio under a larger-the-better condition, the temperature had the highest impact, followed by IL, PcP, and MgSO_4_ as well. The deduced best condition would be 25°C, 1.5 g of TBABr, 15 mg of PcP, and 2.0 g of MgSO_4_. The Taguchi confirmation test was then performed, and it was found that under the above-mentioned best condition, the protein extraction rate and percentage of remaining polysaccharide were 98.6% and 93.5%, respectively.

### 3.2 Preliminary structural analysis of PcP

**FIGURE 3:**
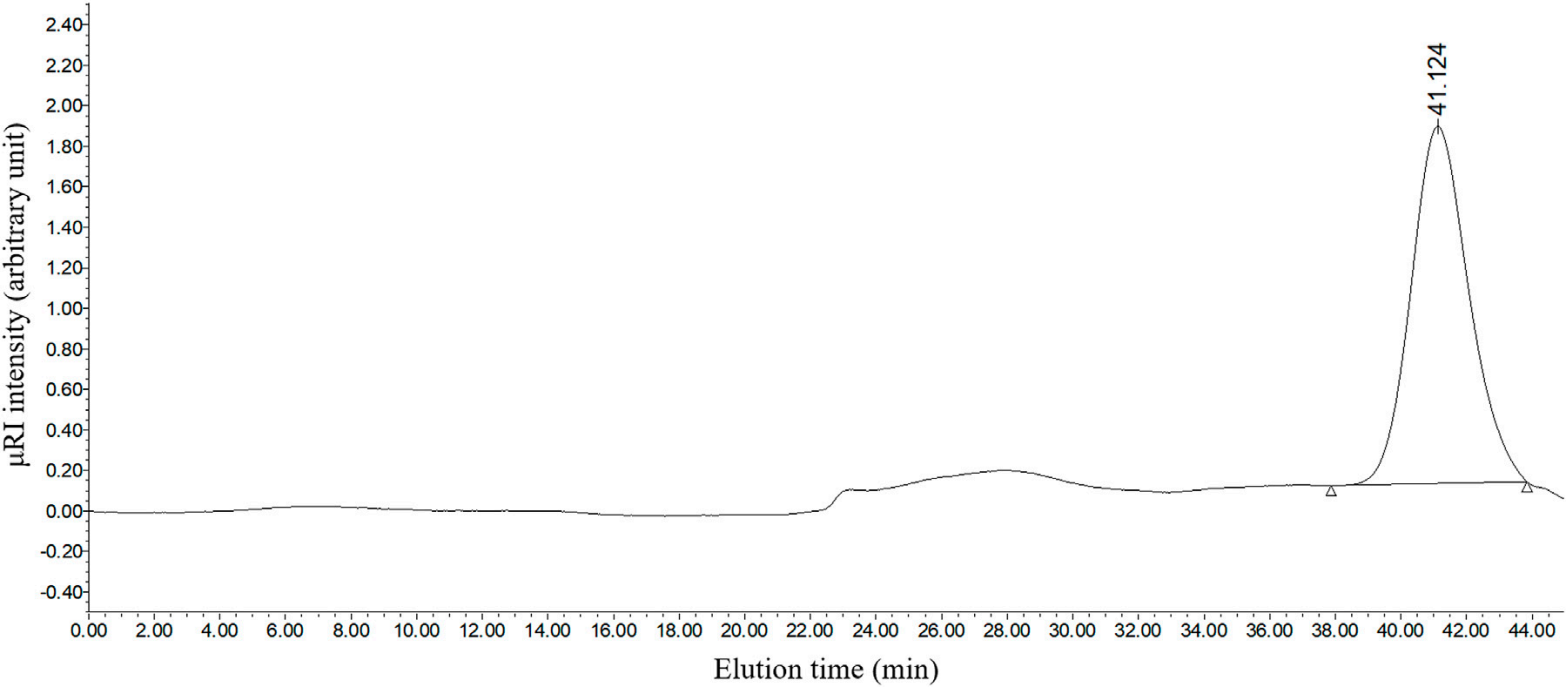
Elution profile of the purified PcP using HPGPC.

Under the best condition, PcP obtained from the aqueous phase was purified by dialysis. The solution was subsequently added to four volumes of absolute ethanol to precipitate the polysaccharide. After centrifugation and freeze-drying, the homogeneity and molecular weight of the processed PcP were determined by HPGPC. The result of HPGPC indicated a single and symmetrical peak with the retention time at 41.124 min, suggesting that the tested sample was homogeneous (Figure 3). The molecular weight of the highest peak (Mp), weight average molecular weight (Mw), number average molecular weight (Mn), and polydispersity index (Mw/Mn) of the purified PcP in the 0.05 M NaCl solution were 6,536 Da, 7,554 Da, 5,467 Da, and 1.38, respectively.

**FIGURE 4:**
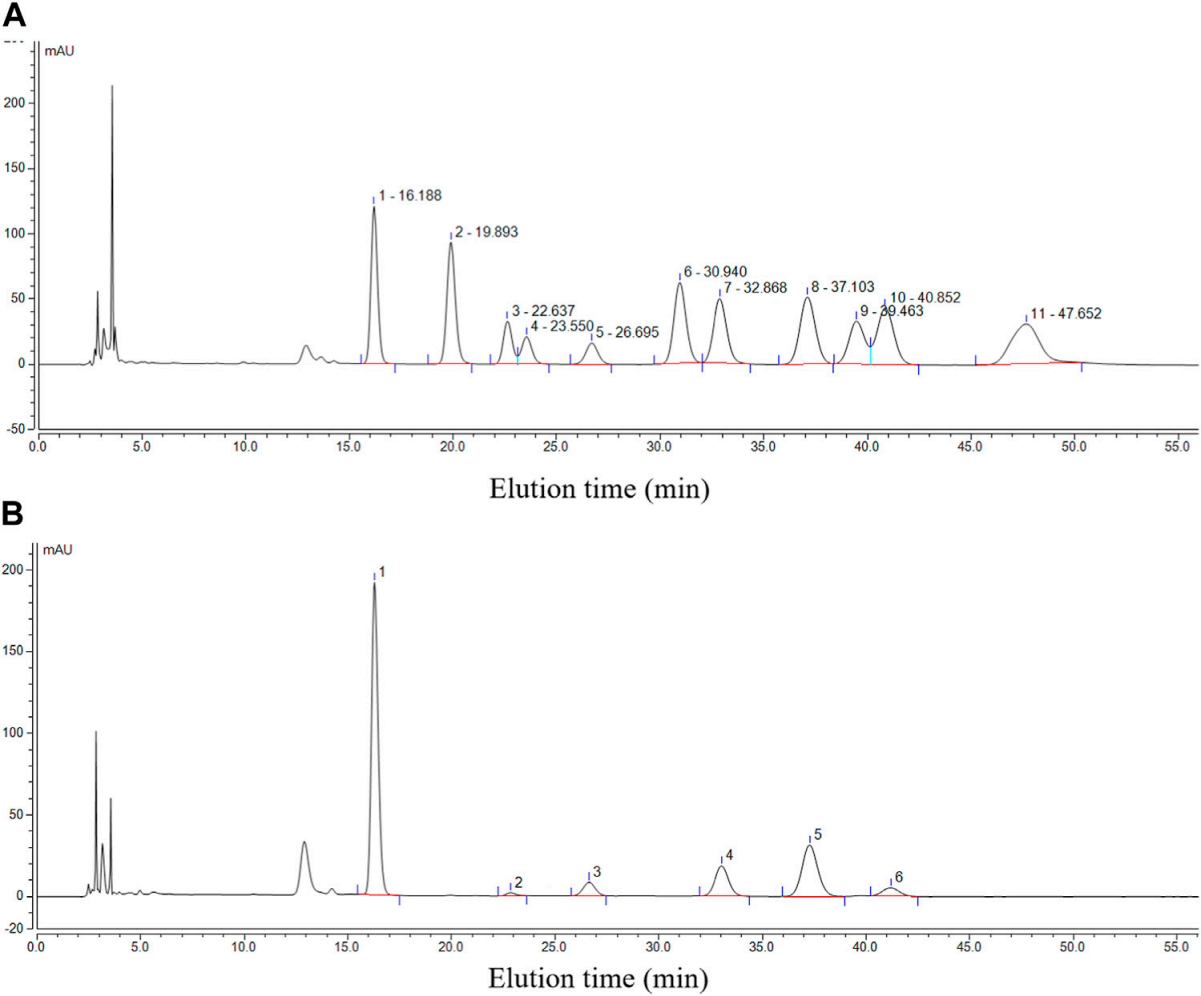
Chromatographic profile of individual monosaccharide standards **(A)** and the purified PcP **(B)**. Note that 1 in B denotes Man, 2 denotes Rha, 3 denotes Gal-UA, 4 denotes Glc, 5 denotes Gal, and 6 denotes Ara.

For monosaccharide composition analysis, reference chromatography was established by using 11 monosaccharide standards, namely, mannose (Man, Rt: 16.188 min), D-glucosamine HCl (GlcN, Rt: 19.893 min), rhamnose (Rha, Rt: 22.637 min), glucuronic acid (Glc-UA, Rt: 23.550 min), galacturonic acid (Gal-UA, Rt: 26.695 min), D-galactosamine HCl (GalN, Rt: 30.940 min), glucose (Glc, Rt: 32.868 min), galactose (Gal, Rt: 37.103 min), xylose (Xyl, Rt: 39.463), arabinose (Ara, Rt: 40.852 min), and fucose (Fuc, Rt: 47.652 min) (Figure 4A). It was found that purified PcP comprised Man, Gal, Glc, Gal-UA, Ara, and Rha with a molar ratio of 33:13:8:3.5:2:1 (Figure 4B). This was in line with the previous report in which Man, Gal, Glc, and Ara were the key monosaccharides in *P. cyrtonema* (Hu et al., 2021).

**FIGURE 5:**
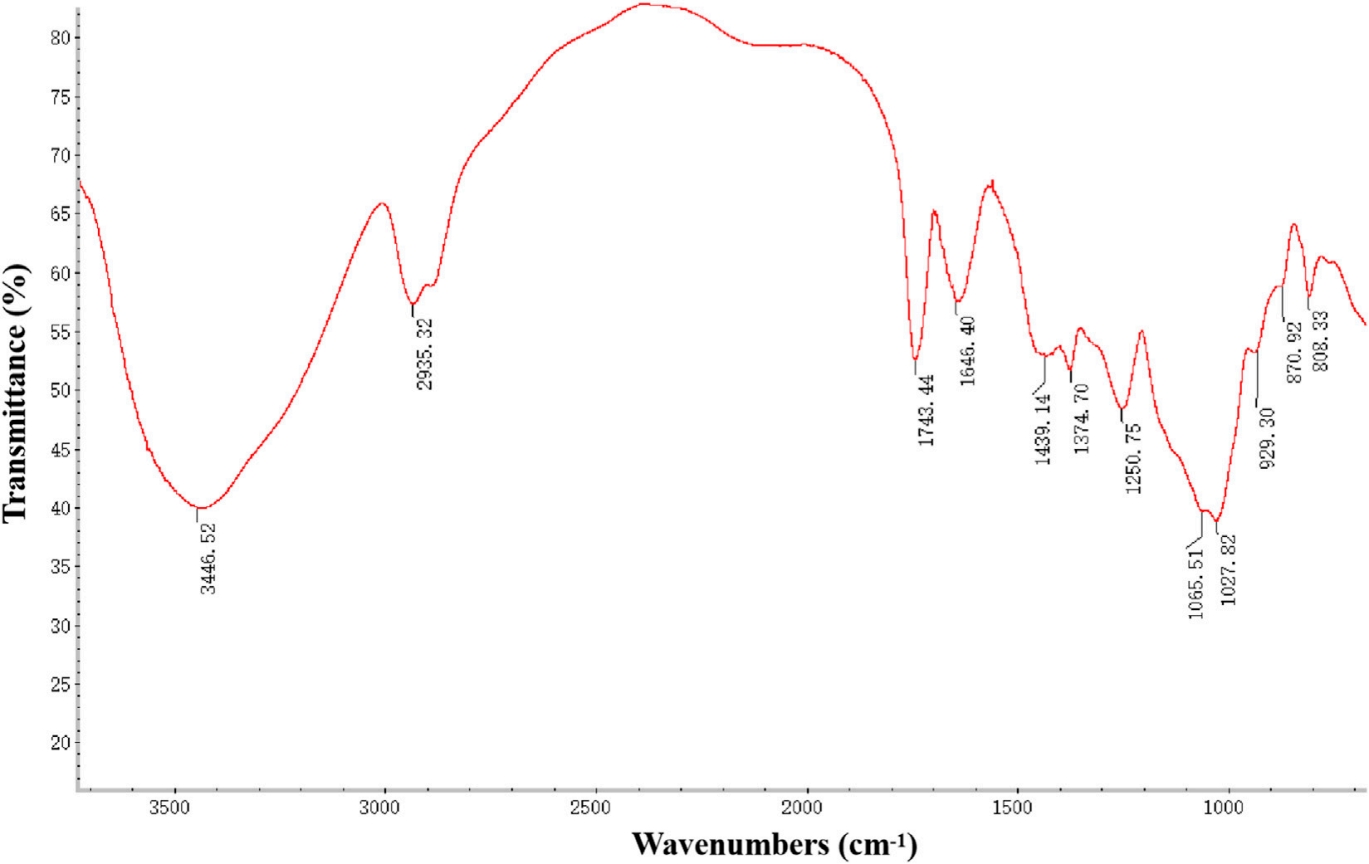
FT-IR spectra of the purified PcP.

In the FT-IR spectra of purified PcP (Figure 5), we identified three typical absorption peaks of polysaccharide. The first peak was a wide stretching peak at 3,446.52 cm^−1^, showing the O-H stretching vibration between or within the polysaccharide molecules. The second peak was a moderate absorption peak at 2,935.32 cm^−1^, indicating the C-H (-CH_3_ and -CH_2_) stretching vibration. The third peak at 1,743.44 cm^−1^ suggested C=O stretching vibration in glucuronic acid molecules. Three peaks at 1,439.14 cm^−1^, 1,374.70 cm^−1^, and 1,250.75 cm^−1^ indicated C-H deviational vibration. Strong absorption peaks at both 1,065.51 cm^−1^ and 1,027.82 cm^−1^ proved once more the existence of pyranoside. The peak at 870.92 cm^−1^ was attributed to the β-type glycosidic linkage in PcP.

## 4 Conclusion

**FIGURE 6:**
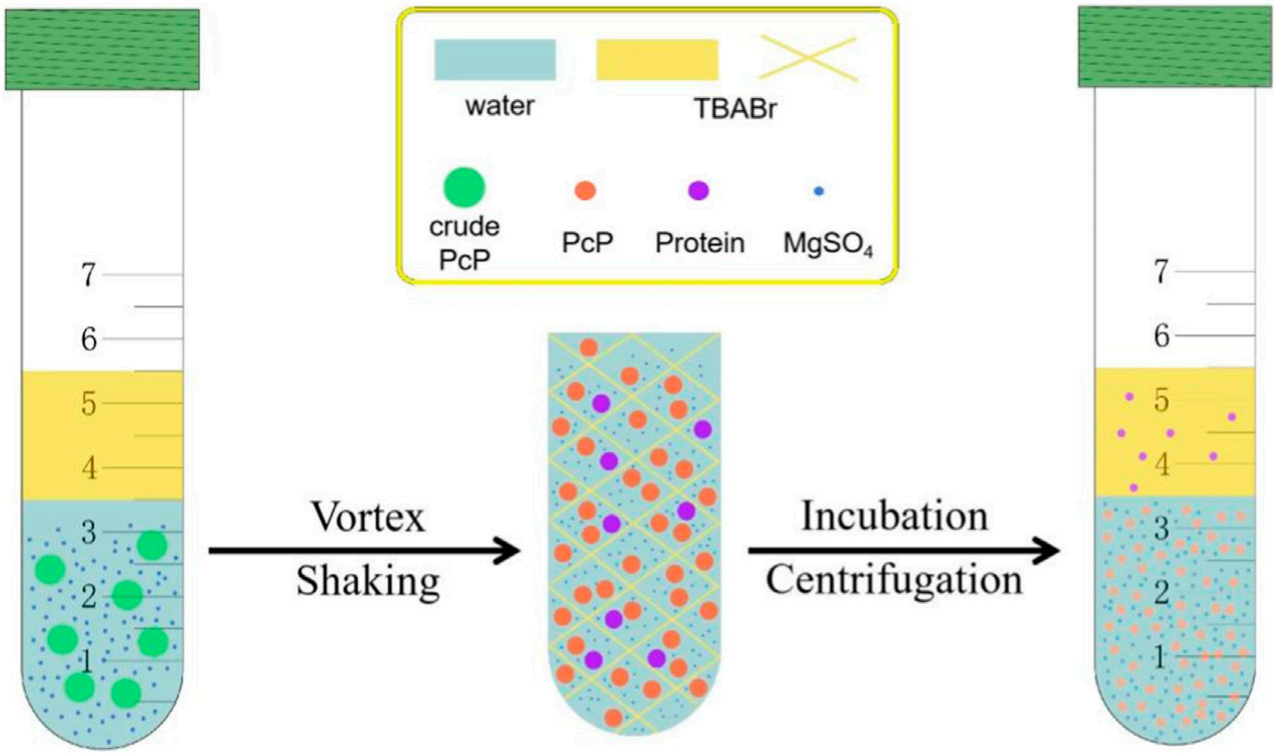
Experimental outline for extracting the protein from crude PcP.

In the ILATPS, after the IL and water are mixed thoroughly by vortexing, water molecules and polysaccharide are bounded together due to hydrogen bonding interactions. Meanwhile, hydrophobic or electrostatic interactions allow the IL to hold on to the protein. By incubation and centrifugation, the carbohydrate and protein are accumulated in the lower water and upper IL phases, respectively (Figure 6). For the past decades, eliminating the protein from crude polysaccharide has been a standard procedure before the purified carbohydrate was subjected to structural determination or bioactivity assessment. Generally, researchers have few options for deproteinization, and the most commonly used approach was the Sevag method, which involved repetitive operations and hazardous organic solvents. The trichloroacetic acid solution was also reported to precipitate the protein (Samrot et al., 2020), but whether acids could influence the properties of carbohydrates merited further investigation. The application of ILs to purify protein from crude polysaccharide offered a “greener” solution due to their remarkable characteristics including negligible vapor pressure and non-flammability under ambient conditions (Yang et al., 2018). To the best of our knowledge, the combination of TBABr and MgSO_4_ has not been used in separating the plant protein and carbohydrate.

Using the TBABr-based partitioning system, the polysaccharide and protein extracted from the stem of *P. cyrtonema* were successfully separated. A Taguchi experiment with an L9 (3^4) orthogonal array was designed to analyze the influence of four parameters concerning the extraction efficiency. Despite various conditions, the protein extraction efficiency was satisfactory (all above 90%). Analyzing the main effect based on S/N ratios suggested that 25°C, 1.5 g of TBABr, 15 mg of PcP, and 2.0 g of MgSO_4_ were the best conditions. A validation experiment proved that the extraction efficiency for protein and polysaccharide in the ILATPS was 98.6% and 93.5%, respectively. This method is environment friendly and easy to handle. It offers a practical tactic to purify polysaccharides of plant origin.

## Data Availability

The raw data supporting the conclusion of this article will be made available by the authors, without undue reservation.
